# STS-NLSP: A Network-Based Label Space Partition Method for Predicting the Specificity of Membrane Transporter Substrates Using a Hybrid Feature of Structural and Semantic Similarity

**DOI:** 10.3389/fbioe.2019.00306

**Published:** 2019-11-06

**Authors:** Xiangeng Wang, Xiaolei Zhu, Mingzhi Ye, Yanjing Wang, Cheng-Dong Li, Yi Xiong, Dong-Qing Wei

**Affiliations:** ^1^State Key Laboratory of Microbial Metabolism, School of Life Sciences and Biotechnology, Joint Laboratory of International Cooperation in Metabolic and Developmental Sciences, Ministry of Education, Shanghai Jiao Tong University, Shanghai, China; ^2^Peng Cheng Laboratory, Shenzhen, China; ^3^School of Sciences, Anhui Agricultural University, Hefei, China

**Keywords:** membrane transporter, substrate specificity, structural fingerprint, chemical ontology, multi-label classification

## Abstract

Membrane transport proteins play crucial roles in the pharmacokinetics of substrate drugs, the drug resistance in cancer and are vital to the process of drug discovery, development and anti-cancer therapeutics. However, experimental methods to profile a substrate drug against a panel of transporters to determine its specificity are labor intensive and time consuming. In this article, we aim to develop an *in silico* multi-label classification approach to predict whether a substrate can specifically recognize one of the 13 categories of drug transporters ranging from ATP-binding cassette to solute carrier families using both structural fingerprints and chemical ontologies information of substrates. The data-driven network-based label space partition (NLSP) method was utilized to construct the model based on a hybrid of similarity-based feature by the integration of 2D fingerprint and semantic similarity. This method builds predictors for each label cluster (possibly intersecting) detected by community detection algorithms and takes union of label sets for a compound as final prediction. NLSP lies into the ensembles of multi-label classifier category in multi-label learning field. We utilized Cramér's V statistics to quantify the label correlations and depicted them via a heatmap. The jackknife tests and iterative stratification based cross-validation method were adopted on a benchmark dataset to evaluate the prediction performance of the proposed models both in multi-label and label-wise manner. Compared with other powerful multi-label methods, ML-*k*NN, MTSVM, and RA*k*EL*d*, our multi-label classification model of NLPS-RF (random forest-based NLSP) has proven to be a feasible and effective model, and performed satisfactorily in the predictive task of transporter-substrate specificity. The idea behind NLSP method is intriguing and the power of NLSP remains to be explored for the multi-label learning problems in bioinformatics. The benchmark dataset, intermediate results and python code which can fully reproduce our experiments and results are available at https://github.com/dqwei-lab/STS.

## Introduction

Membrane transport proteins, also known as transporters or carriers, are a diverse and large group of proteins that transport various hydrophilic molecules, encompassing ions and small molecules across lipid bilayers within a cell or between cells, thus playing crucial roles in various biological functions, such as binding with small molecules in extracellular space, which is the key component to determine the bioavailability and biological activity of chemicals, i.e., their adverse and therapeutic effects (International Transporter et al., 2010). In recent years, a number of efflux and influx transporters from ATP binding cassette (ABC) (Chen et al., 2016) and solute carrier (SLC) (Nyquist et al., 2017) families have attracted significant interest, since they are of vital importance in determining the ADMET (absorption, distribution, metabolism, excretion, and toxicity) properties of a wide range of drugs and xenobiotics. More importantly, membrane proteins are the major media of multi-drug resistance in cancer (Szakács et al., 2006; Fletcher et al., 2010). For example, multi-drug resistance protein 1 (MDR1; aka P-glycoprotein and ABCB1) is overexpressed in many malignant neoplasms and its expression can also be induced by chemotherapy. The overexpression of MDR1 has proven to be correlated with drug resistance in breast, prostate and lung cancer (Holohan et al., 2013). To make things worse, widely-applied targeted drugs such as nilotinib, imatinib, sunitinib, and erlotinib are also identified as regulators and substrates for specific transporters. Thus, understanding the specificity of transporter substrates (identification of potential transporters for existing and novel drug molecules at the early phase of drug discovery process) is not only momentous to the discovery and development of safe and efficacious drugs but also helpful to identify potential drug resistance in anti-cancer therapeutics. However, experimental methods to profile compounds against a panel of transporters are time- and resource-consuming. It should be of high value to develop *in silico* classification models to predict the specificity of membrane transporter substrates.

Generally, two major categories of computational approaches are utilized to predict potential transporters involved in membrane transport of chemicals (Shaikh et al., 2017). The first type of approaches are receptor-based methods, which evaluate the interaction details between transporters and drug molecules via available three-dimensional structures of macromolecules. However, these approaches are hindered by the scarcity of the high-resolution structures of membrane transporters, which are generally difficult to be resolved by experimental technologies. The second category of approaches are ligand-based methods (Chakraborty et al., 2017), via the structural likeness of ligands to known substrates. The most commonly applied ligand-based approach is the quantitative structure-activity relationship (SAR or QSAR) model, which aims to build a mapping from molecular descriptors of ligands to biological functions (e.g., whether the compound is a specific transporter substrate). Many SAR or QSAR models have been built to classify substrates and non-substrates for a specific type of transporters, such as P-glycoprotein (P-gp/MDR1/ABCB1) (Huang et al., 2007; Wang et al., 2011; Poongavanam et al., 2012; Li et al., 2014), BCRP/ABCG2 (Zhong et al., 2011; Hazai et al., 2013; Gantner et al., 2017), MRP1/ABCC1 (Lingineni et al., 2017) by a variety of machine learning models, including linear models, neural networks, support vector machines (SVM), and etc. Li et al. (2014) developed the naïve Bayesian classifier to predict potential P-gp substrates using simple molecular properties, topological descriptors, and structural fingerprints on a compiled dataset of 423 P-gp substrates and 399 non-substrates. Hazai et al. (2013) developed an SVM classification model for prediction of BCRP substrates on a dataset composed of 164 BCRP substrates and 99 non-substrates.

However, these traditional QSAR models only consider a single type of carrier at a time. With the ever-accumulating high-quality data of various drug transporters, it is superior to assign a compound into the maximum possible number of transporters. The failure of clinical trials on MDR1 inhibitors such as tariquidar (Pusztai et al., 2005) and zosuquidar (Cripe et al., 2010) also suggests that, in order to block the potential drug efflux of cancer cell entirely, we need to consider the specificity of as much transporters as possible in the design phase of new drugs. Thanks to the efforts conducted by Mak et al. (2015), the interaction data on various types of transporters and their substrates and modulators were curated on Metrabase database exploited for QSAR modeling. In addition to the data from Metrabase, Shaikh et al. (2017) further retrieved data of ABCG2, MDR1 and MRP1 from the literature, to construct a benchmark dataset of substrates and non-substrates of the 13 transporters from ABC and SLC families. In their recent study (Shaikh et al., 2017), they employed proteochemometric (PCM) modeling technique to enable simultaneous consideration of multiple transporters. They built PCM- and QSAR-based predictive models for the transporter-substrate specificity of pharmaceutically important membrane transporters. In those models, the physicochemical, topological descriptors of ligand molecules, MACCS and variants of Morgan fingerprints were used as input features.

Inspired by the successful application of multi-label classification systems in the classification of drugs (Chen et al., 2014), we formulated the problem of transporter-substrate specificity as a multi-label classification task since some compounds can be substrates of more than one transporters. Typically, multi-label classification (MLC) models are divided into three major groups: algorithm adaptation, problem transformation, and ensembles of multi-label classifier (EMLC). Algorithm adaptation methods incorporate specific tricks that convert traditional single-label learning classifiers into multi-label ones. The representative model of this group is ML-*k*NN (Zhang and Zhou, 2005). For the problem transformation method, it converts multi-label learning tasks into one or several single-label problems. For example, label powerset (LP) is a method of problem transformation, which trains models on each possible subset of label sets (Gibaja and Ventura, 2014). For a dataset with high cardinality in label set, LP is prone to overfitting because of the exponentially increased number of subsets. To tackle the overfitting nature of label powerset, Tsoumakas et al. (2011) try to segment the label space into subspaces and apply label powerset in these subspaces. They proposed the RA*k*EL*d* method, which cuts the label set into *k* disjoint subsets. One major drawback of RA*k*EL*d* is that the *k* is arbitrarily chosen without incorporating the label correlations which can be possibly learnt from training data. The Network-based Label Space Partition (NLSP) (Szymanski et al., 2016) is an EMLC built upon LP, and it divides the label sets into *n* small-sized label sets (possibly intersecting) by community detection method which can incorporate the label correlation structures in training set, such that learning *k* representative LP classifiers. As a result, NLSP tackles much less subsets compared to LP and selects *k* in a data-driven manner. For a more detailed explanation of multi-label learning, refer to Zhang and Zhou (2014), Moyano et al. (2018).

**Figure 1:**
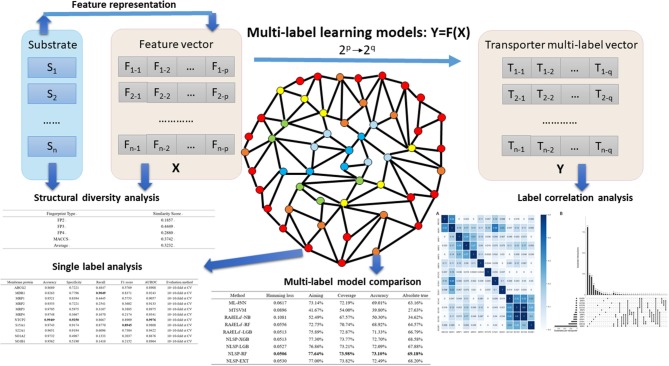
Major steps in the article. Substrates, which were confirmed structural diverse, were featurized into numerical vectors, combined with corresponding transporter multi-label vectors, and then fed into different multi-label learning models. Label correlation analysis provided us insights on the interaction among transporters. To facilitate researchers working on specific membrane transporter, NLSP-RF, with consistently better multi-label performance metrics, was selected after multi-label model comparison for the transporter-wise (single label) analysis. For more detailed description, refer to the subsequent parts in this article.

In the present study, we developed an *in-silico* method for predicting the Specificity of membrane Transporter Substrates based on the Network-based Label Space Partition algorithm, termed STS-NLSP, which has both unleashed the correlation among labels and integrated two types of similarity-based features. Specifically, a given compound substrate was classified as one or more of the following classes of transporters (Shaikh et al., 2017): (i) ABCG2; (ii) MDR1; (iii) MRP1; (iv) MRP2; (v) MRP3; (vi) MRP4; (vii) NTCP2; (viii) S15A1; (ix) S22A1; (x) SO1A2; (xi) SO1B1; (xii) SO1B3; (xiii) SO2B1. In order to represent the information of substrates, we not only used the structural fingerprints, but also employed their biological information (i.e., chemical ontology), extracted from the ChEBI database (Degtyarenko et al., 2008). Then, we compared our NLSP-based methods to three different types of multi-label classification methods constructed on identical features. Our results demonstrated that the NLSP-RF model yielded out consistently better performance than another two types of methods using the jackknife test on the benchmark dataset, and we chose it as our final STS-NLSP. Label-wise analysis, validated via iterative stratification, of the final models was also performed for the convenience of experimental biologists. The major steps in the article are summarized in Figure 1.

## Results and Discussion

### Structural Diversity Analysis

In the total of 1, 846 structural different substrates on the benchmark dataset, we calculated the similarity scores of four types of fingerprints, FP2, FP3, FP4, MACCS, and their average similarity score (SS) for each pair (1, 702,935 different pairs in total) of substrates. The higher the score was between two substrates, the more similar they were each other. Listed in Table 1 were the average values of all pairs for the four type of similarity scores, and the average of these four types. The results demonstrated that the dataset of substrates was structurally different and diverse in terms of 2D fingerprints. We could thus put more confident on the representativeness of this dataset. The average similarity score of FP2 was lowest among the four types of fingerprints. Since the four types of fingerprints presented distinct attributes of the molecules, we used the average similarity score to represent their 2D fingerprint similarity for each pair of substrates.

**Table 1:** The average SS of all pairs of substrates on the benchmark dataset for the four types of fingerprints.

**Fingerprint type**	**Similarity score**
FP2	0.1857
FP3	0.4449
FP4	0.2880
MACCS	0.3742
Average	0.3232

### Label Correlation Analysis

**Figure 2:**
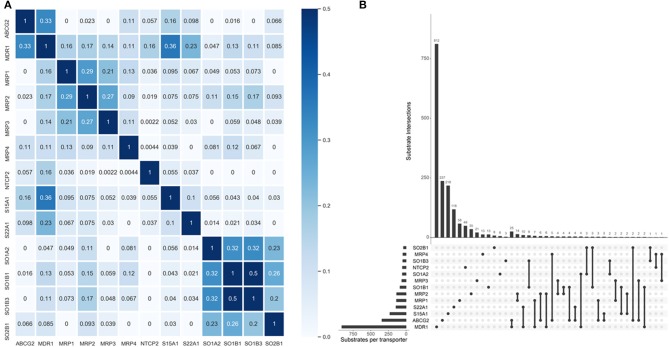
Label correlation landscape. **(A)** The pair-wise heatmap visualization of Cramér's V statistics. **(B)** The UpSet visualization of label intersections. The horizontal bars show the number of substrates per transporter and the vertical bars show the number of substrates per transporter category intersection. The filled dots denote the transporter whose exclusive substrates are counted in the corresponding vertical bars. The vertical lines stand for the intersection of substrates of specific transporters. More dots they encompass, more intersections are considered for the tallying of the corresponding vertical bars.

One primary merit of multi-label learning vis-à-vis single-label learning framework is the explicit utilization of label correlations (Zhang and Zhou, 2014). Bias corrected Cramér's V statistics were calculated for all the possible label pairs and depicted in Figure 2A. The UpSet visualization (Lex et al., 2014) of label-set intersections is shown in Figure 2B. We found 25 substrates are both transported by MDR1 and ABCG2, which is intuitive because MDR1 and ABCG2 are both in the superfamily of ATP-binding cassette transporters. One major common substrate of MDR1 and ABCG2 is gefitinib (Maemondo et al., 2010), which is the first-line targeted chemotherapy agent for non–small-cell lung cancer. Elevated MDR1 and ABCG2 expression has been demonstrated to confer acquired resistance in in EGFR-expressing cancer cells (Chen et al., 2011). The medical implications of co-transport of MDR1 and ABCG2 in cancer has been already noticed by clinicians and basic researchers. We also found several label sets are correlated, especially for SOB1B1 and SOB1B3, of which the Cramér's V statistic is 0.5. Details about the pair-wise intersection numbers of substrates and the pair-wise Cramér's V statistics between all the transporters are shown in Tables S1, S2.

### Multi-Label Model Comparison

**Figure 3:**
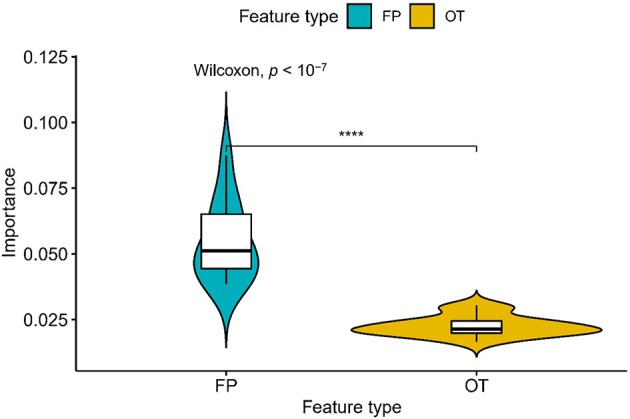
Comparison of feature importance between structural similarity- and semantic similarity-based features. “FP,” fingerprint, stands for structural similarity-based features. “OT,” ontology, stands for semantic similarity-based features. ****p < 0.0001.

We compared the prediction performance of NLSP-based models to another three classification methods (i.e., ML-*k*NN, MTSVM and RA*k*EL*d*-based models) on the identification of specificity of transporter substrates. The classification performances of all the models on the benchmark dataset using jackknife test were shown in Table 2. We found NLSP-RF (random forest-based NLSP) is consistently better than the other models in all the five predefined multi-label measures. On the other hand, we found all the NLSP-based methods perform consistently better than other models, and the MTSVM is the most unsatisfactory model. For the RA*k*EL*d*-based methods, we found the choice of base-learners will have huge impact on the model performance. Therefore, we selected the NLSP-RF as the classification engine to construct the final prediction model. To get deeper insights of this predictive task, we compared the mean feature importance (Gini index) of structural similarity- and semantic similarity-based features on the final prediction model. We found the structural similarity-based features are significantly (*p* < 10^−7^) more important than semantic similarity-based features (Figure 3), suggesting the selectivity of chemicals among different transporters majorly hinges on the 2D structure of chemicals.

**Table 2:** Performance comparison of various multi-label classification methods.

**Method**	**Hamming loss**	**Aiming**	**Coverage**	**Accuracy**	**Absolute true**
ML-*k*NN	0.0617	73.14%	72.19%	69.01%	63.16%
MTSVM	0.0896	41.67%	54.00%	39.80%	27.63%
RA*k*EL*d*-NB	0.1081	52.49%	67.57%	50.30%	34.62%
RA*k*EL*d*-RF	0.0556	72.75%	70.74%	68.92%	64.57%
RA*k*EL*d*-LGB	0.0513	75.89%	72.87%	71.33%	66.79%
NLSP-XGB	0.0513	77.30%	73.77%	72.70%	68.58%
NLSP-LGB	0.0527	76.86%	73.21%	72.09%	67.88%
NLSP-RF	**0.0506**	**77.64%**	**73.98%**	**73.10%**	**69.18%**
NLSP-EXT	0.0530	77.00%	73.82%	72.49%	68.20%

*The bold value stands for the best value of specific metrics in these models*.

### Single-Label Analysis

As for experimental biologist working on specific membrane protein, it is useful to evaluate multi-label learning models for each label respectively (Michielan et al., 2009; Mayr et al., 2016). We utilized the hyperparameters of the best-performing multi-label model of NLSP-RF and performed 10 times repeated 10-fold stratified cross validation (10 ×10-fold st CV) (Sechidis et al., 2011), because the jackknife test is rather time-consuming and tends to overestimate different performance measures (Kohavi, 1995), The details are listed in Table 3. We found NLSP-RF perform well in all the single-label subtasks from the viewpoint of accuracy and AUROC, but perform worse in the prediction subtask of MRP2, MRP3, MRP4, SO1A2, SOB1B1 in view of F1 score, which is intuitive because our benchmark dataset is highly-imbalanced for these five proteins. We also compared our model with the previous results from Shaikh et al. (2017). Although we did not manually collect equal-sized negative data for each transporter, our model performs similarly well except for the subtasks suffering from imbalance learning problem.

**Table 3:** Label-wise analysis of best-performing multi-label learning model.

**Membrane protein**	**Accuracy**	**Specificity**	**Sensitivity**	**CCR**	**F1 score**	**AUROC**	**Evaluation method**
ABCG2	0.8689	0.7221	0.4847	0.6034	0.5769	0.8908	10 ×10-fold st CV
MDR1	0.8263	0.7796	**0.9049**	0.8422	0.8371	0.9243	10 ×10-fold st CV
MRP1	0.9521	0.8394	0.4445	0.6419	0.5753	0.9057	10 ×10-fold st CV
MRP2	0.9353	0.7221	0.2541	0.4881	0.3602	0.9133	10 ×10-fold st CV
MRP3	0.9705	0.5975	0.3107	0.4541	0.3885	0.8975	10 ×10-fold st CV
MRP4	0.9748	0.3667	0.1670	0.2668	0.2174	0.9341	10 ×10-fold st CV
NTCP2	**0.9940**^a^	**0.9250**	0.8667	0.8958	0.8909	**0.9976**	10 ×10-fold st CV
S15A1	0.9743	0.9174	0.8770	**0.8972**	**0.8945**	0.9808	10 ×10-fold st CV
S22A1	0.9651	0.9194	0.6096	0.7645	0.7304	0.9422	10 ×10-fold st CV
SO1A2	0.9732	0.4967	0.1333	0.3150	0.2037	0.8676	10 ×10-fold st CV
SO1B1	0.9562	0.5190	0.1410	0.330	0.2152	0.8964	10 ×10-fold st CV
ABCG2	0.76	0.756	0.764	0.76	0.77	Not available	5-fold cv^b^
MDR1	0.776	0.798	0.751	0.775	0.761		5-fold cv^b^
MRP1	0.826	0.844	0.812	0.828	0.841		5-fold cv^b^
MRP2	0.814	0.886	0.746	0.816	0.804		5-fold cv^b^
MRP3	0.869	0.855	0.885	0.87	0.868		5-fold cv^b^
MRP4	0.905	0.857	0.949	0.903	0.914		5-fold cv^b^
NTCP2	0.93	0.93	0.93	0.93	0.93		5-fold cv^b^
S15A1	0.847	0.819	0.869	0.844	0.864		5-fold cv^b^
S22A1	0.844	0.875	0.813	0.844	0.84		5-fold cv^b^
SO1A2	0.711	0.979	0.419	0.699	0.581		5-fold cv^b^
SO1B1	0.776	0.726	0.829	0.777	0.784		5-fold cv^b^

a *The bold value stands for the best value of specific metrics in the model of NLSP-RF*.

b *5-fold cv results are from Shaikh et al. (2017)*.

### Comparison With Previous Studies

In this article, the benchmark dataset proposed by Shaikh et al. (2017) was compiled and implemented to test our multi-label classification method. The differences between our method and Shaikh's method (Shaikh et al., 2017) were summarized in Table 4. To our best knowledge, it is the first study incorporating the prediction of the specificity of membrane transporter substrates into multi-label learning framework, whereas previously published methods were constructed as single-label systems. Compared with the single-label systems, it is much trickier to develop predictive models within multi-label learning framework. In the single-label systems, a balanced dataset of substrates (positive samples) and non-substrates (negative samples) were usually constructed for each single transporter, which can result in overestimated prediction performance than the actual cases where the number of substrates is significantly lower than that of non-substrates. It has been noticed that an increasing number of compounds are simultaneously assigned as substrates of multiple (two or more different) transporters. Using the multi-label system, our model extends the discriminative classes from 1 to 13 at a time.

**Table 4:** Methodological differences between Shaikh's method, and our present method (STS-NLSP).

**Difference**	**Shaikh's method (Shaikh et al., 2017)**	**STS-NLSP**
Learning framework	Single-label learning	Multi-label learning
Machine learning method	SVM, random forest, etc.	NLSP
Dataset distribution	A balanced number of substrates and non-substrates for each single transporter, respectively	Substrates categorized into 13 transporters with an imbalanced distribution (910 substrates for a majority of transporter MDR1, and 39 substrates for a minority of transporter SO2B1)
Features	Molecular descriptors, molecular fingerprints and Sequence-based descriptors for transporter proteins	Average similarity score fingerprints, and semantic similarity
Evaluation metrics	Recall, Specificity, Precision, Accuracy, F1 score, MCC	Aiming, Coverage, Accuracy, Absolute True, Absolute False
Validation method	Five-fold cross validation and independent test using an unseen external set	Jackknife test

Although it is much more complicated and challenging to deal with, our proposed model based on the multi-label system has two main advantages. Firstly, it can simultaneously predict multiple transporters of a given compound as the substrate. Secondly, it does not need prepare the datasets of non-substrates for each single transporter, as the single-label system does, because one positive instance of one transporter could possibly be a negative sample for another. Especially, the single-label systems will take a lot of labor work to manually collect the same number of non-substrates with the increasing available substrates. The multi-label systems can avoid the labor work to build the datasets of non-substrates due to its innate negative nature among labelset. We believe that the multi-label system proposed in our study will further benefit the research about the specificity of membrane transporter substrates, especially for the drug resistance screening in cancer research.

## Materials and Methods

### Benchmark Dataset

We utilized the same benchmark dataset proposed by Shaikh et al. (2017) to evaluate the performance of the proposed models, which contains 2,293 small molecules classified into 13 main classes of transporter substrates. The chemical structures of those small molecules were identified by Simplified Molecular Input Line Entry Specification (SMILES). The detailed composition of the benchmark dataset was listed in Table 5. Thus, the benchmark dataset 𝕊 can be formulated as

(1) $$\begin{array}{l} {𝕊=𝕊}_{1}\cup {𝕊}_{2}\cup \ldots \cup {𝕊}_{i}\cup \ldots \cup {𝕊}_{12}\cup {𝕊}_{13} \end{array}$$

where the subset 𝕊_*i*_ includes the samples from the *i*-th transporter (*i* = 1, 2, …,13), and ∪ stands for the symbol for “union” in the set theory.

**Table 5:** Anatomy of the benchmark dataset 𝕊 according to the 13 classes of transporter substrates (see Equation 1). See Supporting Information for further explanation.

**Subset**	**Name**	**Description**	**Substrates**
𝕊_1_	ABCG2	ATP-binding cassette subfamily G member 2 (BCRP)	344
𝕊_2_	MDR1	Multidrug resistance protein 1 (P-glycoprotein 1)	910
𝕊_3_	MRP1	Multidrug resistance-associated protein 1	138
𝕊_4_	MRP2	Multidrug resistance-associated protein 2	136
𝕊_5_	MRP3	Multidrug resistance-associated protein 3	63
𝕊_6_	MRP4	Multidrug resistance-associated protein 4	47
𝕊_7_	NTCP2	Sodium/taurocholate cotransporter	53
𝕊_8_	S15A1	Solute carrier family 15 member 1 (peptide transporter 1)	230
𝕊_9_	S22A1	Solute carrier family 22 member 1 (organic cation transporter 1)	144
𝕊_10_	SO1A2	Solute carrier organic anion transporter family member 1A2	54
𝕊_11_	SO1B1	Solute carrier organic anion transporter family member 1B1	87
𝕊_12_	SO1B3	Solute carrier organic anion transporter family member 1B3	48
𝕊_13_	SO2B1	Solute carrier organic anion transporter family member 2B1	39
Number of total virtual substrates			2,293^a^
Number of total structural different substrates			1,846^b^

a *The number of virtual substrates is calculated as follows: for a structurally same substrate, its contribution to the total number of virtual substrates is 2 if it occurs in two different classes of transporter substrates; that is 3 if it occurs in three different classes of transporter substrates; and so forth*.

b *Of the 1,846 structural different substrates, 1,591 belong to one class, 145 to two classes, 62 to three classes, 28 to four classes, 12 to five classes, and 4 to six classes, 3 to seven classes, and 1 to nine classes. Refer to Supporting Information for elaborated information of substrates listed in each of 13 classes*.

### Measuring Label Correlation

In order to evaluate the association between two labels, we calculated the bias corrected Cramér's V statistic for all the label pairs (Bergsma, 2013). Cramér's V (also referred to as Cramér's phi, denoted as ϕ_c_) statistic is a measure of association between two categorical variables, ranging from 0 to 1 (inclusive). But it is shown that sample Cramér's V tends to overestimate the correlation compared to its population counterpart (Bergsma, 2013). The bias corrected Cramér's V statistic is given by (here *n* denotes sample size and χ^2^ stands for the chi-square statistic without a continuity correction for a contingency table with *r* rows and *c* columns).

(2) $$\begin{array}{l} \tilde{V}=\sqrt{\frac{{\tilde{\varphi }}^{2}}{\tilde{m}}\;} \end{array}$$

where

(3) $$\begin{array}{l} {\tilde{\varphi }}^{2}=\max\left(0,\varphi^{2}-\frac{\left(r-1\right)\left(c-1\right)}{n-1}\right)\;, \end{array}$$

(4) $$\begin{array}{lll} \varphi^{2} & = & \frac{\chi^{2}}{n} \end{array}$$

and

(5) $$\begin{array}{l} \tilde{m}=\min\left(\tilde{r}-1,\tilde{c}-1\right)\;, \end{array}$$

(6) $$\begin{array}{l} \tilde{r}=r-\left(\frac{{\left(r-1\right)}^{2}}{n-1}\right)\;, \end{array}$$

(7) $$\begin{array}{l} \tilde{c}=c-\left(\frac{{\left(c-1\right)}^{2}}{n-1}\right)\;. \end{array}$$

### Feature Representation

We are to describe the effective formulization of samples in the training and testing datasets in this section. Now, let us address this from both structural and biological (i.e., chemical ontology) angles.

#### Features to Reflect Structural Similarity

The simple 2D fingerprint was chosen to represent the structural characteristics of small molecules, since it not only has high efficiency on the measurement of inter-molecular structural similarity, but also it has achieved effectiveness in similarity search, virtual screening and QSAR studies, despite its neglect of information about the target-ligand interactions, in comparison to 3D shape and docking methods (Duan et al., 2010; Xiao et al., 2013). In this study, four different types of fingerprints were generated by Open Babel (O'Boyle et al., 2011), which are MACCS, FP2, FP3, and FP4, on the basis of SMILES for each substrate. These fingerprints were binary strings, which encode the presence or absence of sub-structural fragments. Given two substrates, their fingerprint similarity was defined by Tanimoto coefficient (Keum et al., 2016),

(8) $$\begin{array}{l} TC=\frac{c}{a+b-c} \end{array}$$

where *a* and *b* are the number of bits set in substrate bit-strings, *c* strands for the number of bits shared by two substrates. The structural similarity score between any pair of two substrates was calculated by the average Tanimoto coefficients of the four types of fingerprints between them. A specific sample is formulated as a 13-D vector via its maximum structural similarity score with those in each of the 13 subsets,

(9) $$\begin{array}{l} D^{StrSim}={\left[\alpha_{1}\;\alpha_{2}{\;\alpha }_{3}\;\ldots {\;\alpha }_{13}\right]}^{T} \end{array}$$

where α_1_ denotes its maximum structural similarity score with the substrates in the subset 𝕊_1_, α_2_ for that in the subset 𝕊_2_, and so on.

#### Features to Reflect Semantic Similarity

In the present study, we utilized the ontology information of compounds, named as ChEBI ontology (Degtyarenko et al., 2008), which was similar to gene ontology, to incorporate the semantic information. ChEBI provides an ontology database of chemical entities with curated biological annotations. The ChEBI ontology information was retrieved from ftp://ftp.ebi.ac.uk/pub/databases/chebi/ontology/ (“chebi.obo,” July 2017). Theoretically, ontologies are limited vocabularies can be conceived as graph structures consisting of “terms” forming the node set and “relations” of two terms forming the edge set. It consists of three separate subontologies, of which the roots will be “chemical entity,” “role,” and “subatomic particle,” respectively (Hastings et al., 2013). As has been stated in a series of studies (Pesquita et al., 2009; Ferreira and Couto, 2010; Couto and Silva, 2011; Couto and Pinto, 2013), there are various ways to measure semantic similarity relying on information content (IC) between two entities based on an ontology. Given any compound which corresponds to a term *c* on the ChEBI ontology, let *p*(*c*) be the usage frequency of the term *c* in some corpus. The information content of a term can be given by

(10) $$\begin{array}{l} IC\left(c\right)=-logp\left(c\right)\; \end{array}$$

given two compounds *c*_1_ and *c*_2_, the following formula was used to measure the semantic similarity between them:

(11) $$\begin{array}{l} sim_{Lin}\left(c_{1},\;c_{2}\right)=\frac{2\times IC\left(c_{MICA}\right)}{IC\left(c_{1}\right)+IC\left(c_{2}\right)} \end{array}$$

where *MICA* is their most informative common ancestor of both *c*_1_ and *c*_2_. A specific sample is formulated as a 13-D vector via its maximum semantic similarity score with those in each of the 13 subsets.

(12) $$\begin{array}{l} D^{SemSim}={\left[\beta_{1}{\;\beta }_{2}{\;\beta }_{3}\;\ldots \;\beta_{13}\right]}^{T} \end{array}$$

where β_1_ means its maximum semantic similarity score with the substrates in the subset 𝕊_1_, β_2_ for that in the subset 𝕊_2_, and so on.

### Multi-Label Classification Methods

#### Network Based Label Space Partition

The NLSP is a newly proposed multi-label learning method and has achieved top performance in many predictive tasks (Szymanski et al., 2016). This method has also recently reached the top performance in the drug classification and enzyme-substrate selectivity prediction tasks by our group (Shan et al., 2019; Wang et al., 2019). Inspired by these current advances, we adopted the data-driven NLSP method for the prediction of specificity of membrane transporter substrates. NLSP divides the predictive modeling into training and classification phase

The training phase is divided into four parts. We firstly establish a label co-occurrence graph on the training set, which can be weighted or not. Then we detect the community on the label co-occurrence graph. There are various community detection algorithms. In this study, we utilized the largest modularity using incremental greedy search (Blondel et al., 2008) method and multiple async label propagation (Raghavan et al., 2007) to fulfill this task. Thirdly, for each community, a corresponding training set is generated by selecting the original dataset with label columns presented in the community. Finally, for each community, a base predictor is learnt on the training set. In this study, we compared the performance of five types of base predictors:

1. **Extremely randomized trees (ERT)** (Geurts et al., 2006) is a tree-based ensemble method that adds more randomness compared to random forests by the random top-down splitting of trees instead of computing the locally optimal cut-point for each feature under consideration. This increase in randomness reduces the variance of the model a bit, at the expense of a slightly greater increase in bias.
2. **Random forests (RF)** (Breiman, 2001; Manavalan et al., 2014, 2018b; Lv et al., 2019; Ru et al., 2019) is a tree-based ensemble method that combines the probabilistic predictions of a number of decision tree-based classifiers to improve the generalization ability over a single estimator.
3. **Support vector machine (SVM)** (Chang and Lin, 2011; Xiong et al., 2011, 2012; Sun et al., 2014; Manavalan and Lee, 2017; Manavalan et al., 2018d; Zhang et al., 2018; Meng et al., 2019) is a widely used classification algorithm which tries to find the maximum margin hyperplane to divide samples into different classes. Incorporated by kernel trick, this method could handle both linear and no-linear decision boundary.
4. **Extreme gradient boosting (XGB)** (Chen and Guestrin, 2016) is a newly proposed boosting method, which has achieved state-of-the-art performance on many tasks with tabular training data (Chen et al., 2018). Traditional gradient boosting machine is a meta algorithm to build an ensemble strong learner sequentially from weak learners such as decision trees s, while XGB is an efficient and distributed implementation of gradient boosting machine.
5. **LightGBM (LGB)** (Ke et al., 2017; Xu et al., 2017; Liao et al., 2018) is another cutting-edge implementation of gradient boosting decision trees. Two innovative techniques, gradient-based one-side sampling and exclusive feature bundling are incorporated in the model training process, which has proven to achieve almost similar accuracy as XGB with up to over 20 times speed-up.

In the classification phase, we just perform predication on all the communities identified in the training phase and fetch the union of assigned labels. For more technical details refer to Szymanski et al. (2016).

#### Benchmark Methods

Inspired by the recent study (Cheng et al., 2017), we compared NLSP-base methods with another three cutting-edge multi-label classification methods, ML-*k*NN (Zhang and Zhou, 2007), MLTSVM (Chen et al., 2016) and RA*k*EL*d*-based methods (Tsoumakas et al., 2011). ML-*k*NN is a lazy learning model based on traditional *k*NN (Fukunaga and Hostetler, 1973). For a new data instance, it firstly finds the top-*k* closest samples in the training set. Secondly, it calculates the number of each label in the *k* samples. Thirdly, based on the aforementioned label number, it estimates the label probability by naïve Bayes method. Finally, the label probability is generated by maximum *a posteriori* estimation. MLTSVM is a variation of twin support vector machine designed for multi-label scenario proposed by Chen et al. (2016). As for twin support vector machine (Khemchandani and Chandra, 2007), it relaxes the parallel constrain of separating hyperplane in SVM thus boosting the training speed (Joachims, 1998). RA*k*EL*d* (RAndom *k* labELsets) is proposed by Tsoumakas et al. (2011) to overcome the overfitting problem of LP method. RA*k*EL*d* divides the label space into *k* disjoint subsets and trains an ensemble of LP classifiers on each subset. Experiments shows that RA*k*EL*d* improves the performance over LP by a considerable margin and is among the best-performing methods especially for application domains with large number of labels (Tsoumakas et al., 2011).

### Model Evaluation Method

The widely applied model validation methods are *k*-fold cross-validation, leave-one-out cross-validation (or called as jackknife test), and independent tests (or called as holdout method) (Chou and Zhang, 1995; Kohavi, 1995; Niu and Zhang, 2017; Han et al., 2018; Zhang et al., 2018; Aparo et al., 2019). Jackknife test uses a single instance from the sample set as the validation data, and the remaining samples as the training data. This process is iterated until each sample in the sample set is used as the validation case.

As for *k*-fold cross-validation (CV), the sample set is segmented randomly into *k* exclusive subsets with equal size. One subset of the *k* subsets is selected as the validation data, and the remnant *k*-1 subsets are as training data. This process is then repeated *k* time, until each of the *k* subsets used as the validation data for one time. A single estimation metric is finally generated by averaging the results from *k* folds. Typically for the classification task, the CV is often performed in stratified manner, which partitions a dataset so that the proportion of samples of each class in each fold equals to that in the whole dataset. Stratified CV is proven to improve CV in terms of bias and variance (Kohavi, 1995). But the Stratified CV for multi-label learning task is male-defined. Experiments on multi-label learning task either utilize presplit training/test set accompanying a benchmark dataset or the unstratified version of cross-validation and holdout method (Madjarov et al., 2012; Zhang and Zhou, 2014). This situation will possible lead to a scenario where the test set is absent of even single positive example of rare labels, causing the zero-divisor problem of various multi-label evaluation metrics. Commonly, researchers avert this problem via the removal of all the rare labels (Heider et al., 2013; Riemenschneider et al., 2016; Xing et al., 2019), which is suboptimal because the rare events are often of greater importance compared to common ones (Taleb, 2007). Two possible interpretations of multi-label stratification exist. One treats the distinct labelsets as unique classes, while another considers each label independently of the rest. The number of distinct labelsets often grow exponentially with the number of labels, which means the first interpretation is not applicable of the task at hand. The next interpretation was thus utilized in this article. Inspired by the study of Sechidis et al. (2011), we utilized 10 times repeated 10-fold iterative stratification cross-validation to validate our best performing multi-label method in a label-wise manner. The basic idea of this method is to iteratively sample each label, respectively in a greed manner. In the whole process, the rare labels are treated in priority to avoid zero-divisor problem and grasp instances with greater importance. The pseudocode of iterative stratification is given by Algorithm 1.

**Algorithm 1:** Iterative Stratification (**D**, *n, r*_1_, …, *r*_*k*_)

	**Input:** A dataset, **D**, consists of a set of labels **L** = {*l*_1_, .., *l*_*q*_}, designated number of folds *k*, required proportion of samples in each fold, *r*_1_, …, *r*_*k*_ (e.g. in 5-fold CV, *k* = 5, *r*_*j*_ = 0.2, *j* = 1… 5) **Output:** Exclusive subsets *S*_1_, …, *S*_*k*_ of **D**
1	// Generate the required number of samples at each fold
2	**for** *j* ← 1 **to** *k* **do**
3	*c*_*j*_ ← |**D**|*r*_*j*_
4	// Generate the required number of samples of each label at each fold
5	**for** *i* ← 1 **to** |*L*| **do**
6	// Calculate the samples of each label in the initial set
7	$D^{i}\leftarrow \left\{\left(D,L\right)\in \text{D}:l_{i}\in L\right\}$
8	for *j* ← 1 **to** *k* **do**
9	$c_{j}\leftarrow |D^{i}|r_{j}$
10	**while** |*D*| > 0 **do**
11	// Identify the label with the fewest (but at least one) remaining samples,
12	// Break ties randomly
13	$D^{i}\leftarrow \left\{\left(D,L\right)\in \text{D}:l_{i}\in L\right\}$
14	$l\leftarrow argmin_{i}\left(\left|D^{i}\right|\right)⋂\left\{i:D^{i}\neq \emptyset \right\}$
15	**foreach** (*D, L*) ∈ *D*^*l*^ **do**
16	// Identify the fold(s) with the largest number of required samples for this label
17	// Break ties by considering the largest number of required samples, break further ties randomly
18	$M\leftarrow {\text{argmax}}_{j=1\ldots k}\left(c_{j}^{i}\right)$
19	**if** |*M*| = 1 **then**
20	*m* ∈ *M*
21	**else**
22	$M^{\prime }\leftarrow argmax_{j\in M}\left(c_{j}\right)$
23	**if** |*M*′| = 1 **then**
24	*m* ∈ *M*′
25	**else**
26	*m* ← *randomElementOf*(*M*′)
27	*S*_*m*_ ← *S*_*m*_⋃{(*D, L*)}
28	*D* ← *D*{(*D, L*)}
29	// Update desired number of examples
30	**foreach** *l*_*i*_ ∈ **L do**
31	$c_{m}^{i}\leftarrow c_{m}^{i}-1$
32	*c*_*m*_ ← *c*_*m*_−1
33	**return** *S*_1_, …, *S*_*k*_

### Performance Metrics for Multi-Label Learning

Multi-label classification algorithms have widely been used in various bioinformatic applications (Zou et al., 2013; Yuan et al., 2016; Wan et al., 2017; You et al., 2018, 2019). Inspired by a set of five metrics established by Chou (2013) and the recommendation of Madjarov et al. (2012), we used the following five metrics to evaluate our multi-label learning model:

(13) $$\begin{array}{l} \left\{ \begin{array}{c} Aiming=\frac{1}{N}\sum_{k=1}^{N}\left(\frac{{∥𝕃}_{k}⋂{𝕃}_{k}^{*}∥}{∥{𝕃}_{k}^{*}∥}\right)\\ Coverage=\frac{1}{N}\sum_{k=1}^{N}\left(\frac{{∥𝕃}_{k}⋂\;{𝕃}_{k}^{*}∥}{∥{𝕃}_{k}∥}\right)\\ Accuracy=\frac{1}{N}\sum_{k=1}^{N}\left(\frac{{∥𝕃}_{k}⋂{𝕃}_{k}^{*}∥}{{∥𝕃}_{k}⋃{𝕃}_{k}^{*}∥}\right)\\ Absolute\;True=\frac{1}{N}\sum_{k=1}^{N}\Delta \left({𝕃}_{k},\;{𝕃}_{k}^{*}\right)\\ Hamming\;loss=\frac{1}{N}\sum_{k=1}^{N}{∥𝕃}_{k}⊝\;{𝕃}_{k}^{*}∥ \end{array}\right. \end{array}$$

where *N* denotes the total number of samples, *M* stands for the total number of labels, ⋃ represents union in set theory and ⋂ represents intersection in set theory, *𝕃*_*k*_ denotes the true label set of *k*-th sample, ${𝕃}_{k}^{*}$ means the predicted label vector of *k*-th sample, ⊝ is the symmetric difference between two sets, and

(14) $$\begin{array}{l} \Delta \left({𝕃}_{k},\;{𝕃}_{k}*\right)=\left\{ \begin{array}{c} 1,\;if\;all\;the\;labels\;in\;{𝕃}_{k}\;equal\;{𝕃}_{k}*\\ 0,\;otherwise \end{array}\right. \end{array}$$

These above metrics have been widely used in bioinformatic applications (Cheng et al., 2017).

### Performance Metrics for Single-Label Learning

Apart from the metrics in the multi-label framework, we also utilized the following metrics to asses our methods in a label-wise manner (He et al., 2018; Manavalan et al., 2018a,c, 2019; Qiao et al., 2018; Xiong et al., 2018, 2019; Xu et al., 2018; Zhang et al., 2018a,b,c, 2019a,b,c; Bian et al., 2019; Su et al., 2019; Wei et al., 2019; Zeng et al., 2019; Zhu et al., 2019; Zou et al., 2019).

(15) $$\begin{array}{l} \left\{ \begin{array}{c} Accuracy=\frac{TP+TN}{TP+TN+FN+FP}\\ Specificity=\;\frac{TN}{TN+FP}\\ Sensitivity=\;\frac{TP}{TP+FN}\\ F1=\;\frac{2}{\frac{1}{\frac{TP}{TP+FP}}+\frac{1}{Sensitivity}}\;\\ CCR=\frac{Sensitivity+Specificity}{2} \end{array}\right. \end{array}$$

where TP, TN, FN, TN are true positives, true negatives, false positives and false negatives for the prediction of each label respectively. In addition, the area under the receive operating characteristic curve (AUROC) were also calculated by trapezoidal rule.

## Conclusion

Accurate prediction of the specificity of substrates for a panel of membrane transporters is of pivotal importance both in the ADMET profiling of drugs and the therapeutics of various cancers. The active drug efflux mediated via transporters lies in the junction of pharmacokinetics and pharmacodynamics. Novel chemicals are impossible to take any effect on cancers if they can be transported out of malignant cells even with satisfactory pharmacokinetic properties and potent *in vivo* anti-cancer activity. In addition, cancer stem cells are characterized by the expression of various transporters, which provides a vicious mechanism enabling cancer recurrence even many years after initial therapy. Identifying compounds without affinity to membrane transporters are prerequisite to the eradication of latent cancer stem cells. The aim of this study is to develop multi-label classification models to predict the classes of transporters given a substrate compound. This method utilized a hybrid of similarity-based features based on structural fingerprints and chemical ontologies. It was shown that the integration of 2D fingerprint and semantic similarity was a feasible and effective way to identify the specificity of a transporter substrate molecule. Various multi-label classification models such as ML-*k*NN, MTSVM, RA*k*EL*d* and NLSP were tested and compared on the benchmark dataset. NLSP-RF was finally selected for constructing the prediction model. To our best knowledge, this article is the first study to apply the multi-label system into the task of predicting of the specificity of membrane transporter substrates.

However, due to the imbalanced nature of classes on the benchmark dataset, our multi-label prediction system preforms unsatisfactory on the proteins of MRP2, MRP3, MRP4, SO1A2, and SOB1B1 in view of F1 score. In the next step, we will make efforts to address the imbalanced datasets via high throughput screens to boost the prediction performance on the specificity of membrane transporter substrates and deploy the optimized final model on a dedicate webserver for clinical and pharmacological usage. Our ultimate objective is to develop pan-transporter inhibitors for anti-cancer therapeutics.

## Data Availability Statement

Publicly available datasets were analyzed in this study. This data can be found here: https://pubs.acs.org/doi/full/10.1021/acs.jcim.6b00508.

## Author Contributions

YX, D-QW, and XW contributed conception and design of the study. XW and YW organized the database. XW, YW, XZ, and MY performed the statistical analysis. XW wrote the first draft of the manuscript. XW, YW, and C-DL wrote sections of the manuscript. All authors contributed to manuscript revision, read, and approved the submitted version.

### Conflict of Interest

The authors declare that the research was conducted in the absence of any commercial or financial relationships that could be construed as a potential conflict of interest.
